# Navigating the Longitudinal Maze: Unravelling Challenges in the Follow‐up of a Dementia Cohort Study

**DOI:** 10.1002/alz.092058

**Published:** 2025-01-03

**Authors:** Sunitha HS, Abhishek Mensegere Lingegodwa, Ajith Partha, Prathima Arvind, Divya NM, Amitha CM, Deva Kumar HS, Rajitha Narayanasamy, Meghana R, Meenakshi Menon, Vindhya Vishwanath, Goutham Velavarajan, Sadhana Singh, Palash K Malo, Albert Stezin, Jonas S. Sundarakumar, Thomas Gregor Issac

**Affiliations:** ^1^ Centre for Brain Research, Indian Institute of Science, Bangalore, Karnataka India; ^2^ Center for Brain Research, IISc, bangalore, Karnataka India; ^3^ Centre for Brain Research, Bangalore, Karnataka India

## Abstract

**Background:**

Dementia, a global health challenge, drives the need for comprehensive understanding. Longitudinal cohort studies are vital, yet maintaining follow‐up in dementia cohorts poses challenges. This study explores challenges in follow‐up, refines protocols, and develops strategies that can elevate dementia research quality.

**Method:**

This is an ongoing longitudinal cohort study on dementia named TLSA (Tata Longitudinal Study of Ageing) in which healthy volunteers are recruited in the community through various strategies such as awareness talks, advertisements, and referrals from other volunteers. All of them undergo a set of clinical assessments, cognitive, blood biochemistry, and MRI examinations at baseline and annual follow‐up visits and are informed about the results of the same by clinicians, thereby maintaining contact regularly. For follow‐ups, each of them is contacted via calls, messages, and Emails once a year. To enlighten them on the findings of research, regular participant meets are conducted, and monthly newsletters are issued. House visits (via mobile unit customized for various assessments) were conducted for participants who were unable to come to the research center but did not want to drop out. This study explored the distinct reasons for dropouts when the volunteers were called for follow‐ups.

**Result:**

The total number of participants is 1484 till December 2023. The total number of dropouts is 113(7.6%). The reasons for dropouts were death 11 (9.7%), unable to contact 35 (30.9%), loss of interest in the study participation 30 (26.5%), relocation 10 (8.8%), health issues 17 (15%), family restrictions 5 (4%), and other unreported reasons 5 (4%).

**Conclusion:**

Enhancing motivation strategies tailored to specific groups such as those who lack interest, have relocated, or face family restrictions can effectively boost follow‐up participation, consequently reducing dropout rates. Tailoring strategies using technology such as social media, infographics, and messaging applications to each group’s unique circumstances can reignite interest in participating in the study, ultimately leading to improved follow‐up rates, and enhancing the overall quality of the research.

